# MiR‐134‐Mbd3 axis regulates the induction of pluripotency

**DOI:** 10.1111/jcmm.12805

**Published:** 2016-02-29

**Authors:** Lei Zhang, Yongchao Zheng, Yuanqing Sun, Ying Zhang, Jia yan, Zhifeng Chen, Hong Jiang

**Affiliations:** ^1^ Department of AnesthesiologyThe Ninth People's HospitalSchool of MedicineShanghai Jiao Tong UniversityShanghaiChina

**Keywords:** miR‐134, Mbd3, induced pluripotent stem cells (iPSCs), neural progenitor cells (NPCs)

## Abstract

MicroRNAs (miRNAs) are post‐transcriptional modulators of gene expression and play an important role in reprogramming process; however, relatively little is known about the underlying regulatory mechanism of miRNAs on how they epigenetically modulate reprogramming and pluripotency. Here, we report that the expression level of microRNA‐134 (miR‐134) was low in mouse embryonic stem cells (mESCs) but significantly up‐regulated during neural differentiation, while down‐regulated during the induction of induced pluripotent stem cells (iPSCs) from neural progenitor cells (NPCs). Inhibition of miR‐134 by miR‐134 sponge promoted the efficiency of reprogramming which also was highly similar to mESCs. On the contrary, up‐regulation of miR‐134 repressed iPSCs induction. We also found that inhibition of miR‐134 promoted the maturation of pre‐iPSCs and increased its pluripotency. We also showed that miR‐134 can directly target to the pluripotency related factor Methyl‐CpG‐binding domain protein 3 (Mdb3) 3′ untranslated regions (3′ UTR) to down‐regulate its expression. And Mbd3 was found to promote the induction of iPSCs and could block the repression of reprogramming caused by overexpression of miR‐134. This work revealed the critical function of miR‐134‐Mbd3 axis on regulating reprogramming and pluripotency of iPSCs derived from the NPCs, and might provide an insight into the miR‐134‐Mbd3 axis on regulating the iPSCs quality for further clinical treatment.

## Introduction

Induced pluripotent stem cells (iPSCs) are a type of embryonic stem cell (ESC)‐like cells which can be produced from mouse and human somatic cells by viral‐mediated transduction of defined pluripotency transcription factors like Oct4, Sox2, Klf4 and c‐Myc 1. As similar to ES cells in terms of morphology, pluripotency gene expression pattern and ability to form teratomas with all three germ layers, iPSCs generated from patient‐specific somatic cells provide promise for personalized cell therapy 2. However, the inefficiency of reprogramming and the low quality of iPSCs limit their potential applications in cell therapy. The efficiency of alkaline phosphatase‐positive (AP+) colony formation with the four Yamanaka's factors (Sox2, Klf4, Oct4, c‐Myc; SKOM) in mouse fibroblasts is about 1% of the starting population, but only around 1 in 10 of those colonies is sufficiently reprogrammed to be chimaera competent 3. In human fibroblasts, only about 0.01% of cells transduced with SKOM form AP+ iPSC colonies 4. The inherently low efficiency of iPSC derivation benefits from selection approaches that distinguish successfully reprogrammed clones from partially reprogrammed or simply transformed colonies 5. While some studies have described techniques for improving the efficiency of iPS cell generation through the use of chemical compounds, different cell types, interfering with the epigenetic status of donor cells, or modulating different signal pathways 3, 6, 7, 8. On the other hand, regarding the safety concerns of iPS cells, strategies of generating integration‐free iPS cells have been developed but the quality of resulting integration‐free iPS cells still needs to be completely characterized 9. Moving towards to the eventual goal of clinical application, overcome limitations such as low reprogramming efficiency, clone quality, and cell maturity is necessary. Some chemical inhibitors involved in DNA methylation, histone methylation and acetylation can improve reprogramming efficiencies and kinetics, besides, other molecules, such as Wnt3a, 2i and A‐83‐01, may alter the signalling states of cells undergoing reprogramming and drive the complete transition into full‐term iPSCs 10. In a recent report, many signalling pathways, transcription factors, epigenetic factors and noncoding RNAs are found to control the reprogramming efficiency and pluripotency of iPSCs 1. But the specific and potential regulatory mechanisms by which other reprogramming factors and molecular networks especially the unknown noncoding RNAs may participates in controlling the reprogramming efficiency and pluripotency of iPSCs will also need to be more discovered.

MicroRNAs (miRNAs) are 20–25 nucleotide (nt) non‐coding RNAs that bind to partially complementary target sites in mRNA 3′ untranslated regions (3′ UTRs) through an imperfect match, which results in degradation of the target mRNAs, or translational repression of the encoded proteins at the post‐transcriptional level 11. An increasing number of studies have demonstrated that miRNAs can regulate the ESCs self‐renewal, even specific to ESCs enhance the production of iPSCs. MiR‐302, which is expressed abundantly in human ESCs, has been implicated in reprogramming able to convert human cancer cell lines to cells that resembled ESCs and Lin28 can actually promote reprogramming by the repression of differentiation induced by the let7 family of microRNAs which has a role in the self‐renewal of breast cancer cells 12. MiR‐145 directly target the Sox2, Oct4, Klf4 to reduce ES cells pluripotency, promoting differentiation cooperated with OCT4 to form a double‐negative feedback loop that switches the hESCs between self‐renewal and differentiation 13. A combination of mature miRNAs (miR‐302s, miR‐200c and miR‐369s) can also reprogramme mouse and human cells to a pluripotent state by using transfection reagents, which may be safer for biomedical research by avoiding the vector‐based gene transfer system 14. But the study of miRNAs regulatory mechanism in iPSCs remains largely unknown, the characterization of the miRNA pathways in regulating reprogramming efficiency and pluripotency of iPSCs needs further investigation.

Mbd3 (Methyl‐CpG binding domain protein 3) is an essential scaffold protein of the NuRD (Nucleosome Remodeling and Deacetylase) complex, which is essential for embryonic development and pluripotent stem cell differentiation 15. Study indicates that Mbd3 is essential for early embryogenesis and it is essential to maintain full mouse ES cell pluripotency 16. Knockdown of Mbd3 in mouse ES cells up‐regulates the expression of Cdx2 which is important for the formation of trophectoderm, and Mbd3 was recruited by GCNF to the Oct4 promoter to repress its expression through DNA methylation in the process of ES cell differentiation 17. Recent report show that the Mbd3/NuRD complex plays a key role in reprogramming in certain contexts and that a chromatin complex required for cell differentiation can also promote reversion back to a naïve pluripotent cell state 15. However, the deep molecular mechanism of Mbd3 in reprogramming and their function in enhancing the reprogramming efficiency and quality are worthy of further investigation, and will be helpful in finding excellent quality and unified iPSCs to facilitate clinical application.

Here, we found that the level of miR‐134 was upregulated during the neuralgenesis from ES cells and downregulated during the reprogramming of neural progenitor cells (NPCs). Inhibition of miR‐134 enhanced the efficiency of reprogramming and promoted the maturation of iPSCs. Our further study showed that miR‐134 regulated the iPSCs generation by directly repressing the Mbd3 by directly targeting the 3′ UTR. These results indicated that the critical function of miR‐134‐Mbd3 axis on regulating reprogramming and pluripotency of iPSCs derived from the NPCs, and might provide an insight into the miR‐134‐Mbd3 axis on regulating the iPSCs quality for further clinical treatment.

## Material and methods

### Teratoma formation and haematoxylin and eosin staining

All animal experiments were approved by the Animal Care Committee of Shanghai Ninth People's Hospital, Shanghai Jiao Tong University School of Medicine. To generate teratomas, iPSCs clones were digested by trypsin to be the cell suspension at the concentration of 3 × 106 cells/200 μl. Cell suspension was injected into NOD‐SCID mice (from the National Resource Center of Mutant Mice Model Animal Research Center).

Teratomas were harvested and fixed in a formaldehyde solution for 24 h before haematoxylin and eosin staining.

### Production of chimeric mice

The iPSCs were injected into the blastocysts, and then implanted the blastocysts into pseudopregnant ICR mice to produce the chimaeras.

### Separation and culture of NPCs

The neural progenitor cells (NPCs) were isolated from the hippocampus tissue in the brain of newborn mice. The tissue was cut up and then filtered using 200 mesh sieves. The cell filtrate was cultured into the DMEM/F12 medium (Gibco, USA) with B27 (10 g/l) and bFGF (20 ug/l) at 37°C in 5% CO_2_. The cells were cultured for 5 days to form the clones. The single clone was then isolated from the dish for expanding the culture of NPCs.

### NPCs reprogramming

Neural progenitor cells were overexpressed by Oct4 (O), Sox2 (S), Klf4 (K), c‐Myc (M) by infecting with pMx‐Oct4, pMx‐Sox2, pMx‐Klf4 and pMx‐cMyc retroviruses. To generate retroviruses, 8 × 10^6^ PLAT‐E cells were seeded in 10 cm dishes, 12 hrs later we transfected the 8 μg of pMx vector, respectively, (OKMS and control vetor) by using FuGENE 6 (Roche) according to manufacturer's instructions. Forty‐eight hours after the infection, the NPCs were culture in the knockout DMEM medium with leukaemia inhibitor factor (lif) and 20% knockout^™^ serum replacement (KSR)(Gibco).

### Construction of miR‐134 sponge vector

miR‐134 sponge were constructed into the pMx vector. The primers of miR‐134 were as follows: PF:5′‐GGATCCCCCCTCTGGTAACCAGTCA CACCGCCCCTCTGGTAACCAGTCACACCGCCCCTCTGGTAACCAGTCACA‐3′; PR:5′‐ AAGCTTTGTGACTGGTTACCAGAGGGGCGGTGTGACTGGTTACCAGAGGGGCGGTGTGACTGGTTACCAGAGGGG‐3′. The primers were mixed and annealed to be the double‐stranded piece of DNA. The fragment of DNA was inserted into the pMx vector.

### Overexpression of MBD3

We generate cDNAs of mouse Mbd3 by inversing transcription from total mRNA. We then amplified the CDS fragment by PCR. We inserted the fragment into the FUGW lentivirus vector. The primers used were as follows: PF:5′‐GGCGAATTCATGGAGCGGAAGAGGTGGG‐3′ (EcoR1 site). PR:5′‐GGCCTCGAGCTACACTCGCTCTGGCTCC‐3′ (Xho1 site).

### Knockdown of MBD3

The shRNA sequence was referenced form the previous study 16.

Mbd3 shRNA: 5′‐AGCCTTCATGGTGACAGAT‐3′;

Mbd3 control shRNA: 5′‐GCGAAGTGCATTGTGTGGC‐3′.

#### Overexpression of miR‐134

The following primers were annealed and inserted into the pLKO.1 vector:

PF:5′‐CCGGTGTGACTGGTTGACCAGAGGGGCTCGAGCCCCTCTGGTCAACCAGTCACATTTTTG‐3′.

PR:5′‐AATTCAAAAATGTGACTGGTTGACCAGAGGGGCTCGAGCCCCTCTGGTCAACCAGTCACA‐3′.

### Construction of luciferase reporter vector

The wild‐type of Mbd3 3′ UTR fragment was amplified from the genomic DNA by the following primers and inserted into the luciferase reporter vector pGL3cM (Promega, Madison, WI, USA).

PF: 5′‐GGCTCAGCCTTGCCTGGACCAG‐3′.

PR: 5′‐GGCTTATTGAAGGAAAGTGACTTCCTGG‐3′.

Mutant 3′ UTR vector was generated by deleting miRNA seed sequence‐binding sites from the pGL3cM‐wild‐type 3′ UTR luciferase reporter vector.

### Transfection and infection

The methods of generating retroviruses encoding reprogramming factors and further infection of NPCs were referenced by previous papers 15.

For transfection, vector or miRNAs were introduced into the cells seeded in plates on the day before transfection using FugeneHD (Roche, USA).

### Western blotting

1 × SDS lysis buffer (Beyotime, China) for cells lysis before electrophoresis. The membrane was incubated by primary Mbd3 antibody (Santa Cruz, CA, USA), GAPDH (sc‐47724, Santa Cruz, CA, USA). The results visualized by enhanced chemiluminescence (ECL) western blotting substrate (Thermo, Waltham, MA, USA).

### Quantitative real‐time PCR (qRT‐PCR)

#### miRNA qRT‐PCR

The total RNA was isolated by using RNAiso plus (Takara, Japan). MiRNA was reverse‐transcribed to cDNA by using the miRNA‐specific stem‐loop reverse‐transcription primer (Ribobio, China). The amount of target gene expression (2^−ΔΔCt^) was normalized *via* the endogenous small nuclear RNA U6 using miRNA‐specific primers (Ribobio). QRT‐PCR reaction conditions were obeyed by instructions of SYBR Green qPCR Mix (BioRad, Hercules, CA, USA).

#### mRNA qRT‐PCR

The cDNA was subsequently reverse‐transcribed from mRNA by M‐MLV Reverse Transcriptase (Takara) from the total RNA. PCR condition included 40 cycles of amplification using the Stratagene Mx3000P system with SYBR Green qPCR Mix (BioRad). Expression of target genes (2^−ΔΔCt^) was normalized against GAPDH.

### Statistical analyses

Student's *t*‐test was used to determined statistical significance. Values were presented as the mean ± standard deviation (S.D.). *P < 0.05, **P < 0.01, ***P < 0.001.

## Results

### Inhibition of miR‐134 facilitates the initiation of iPSCs generation from Neural Progenitor Cells

**Figure 1:**
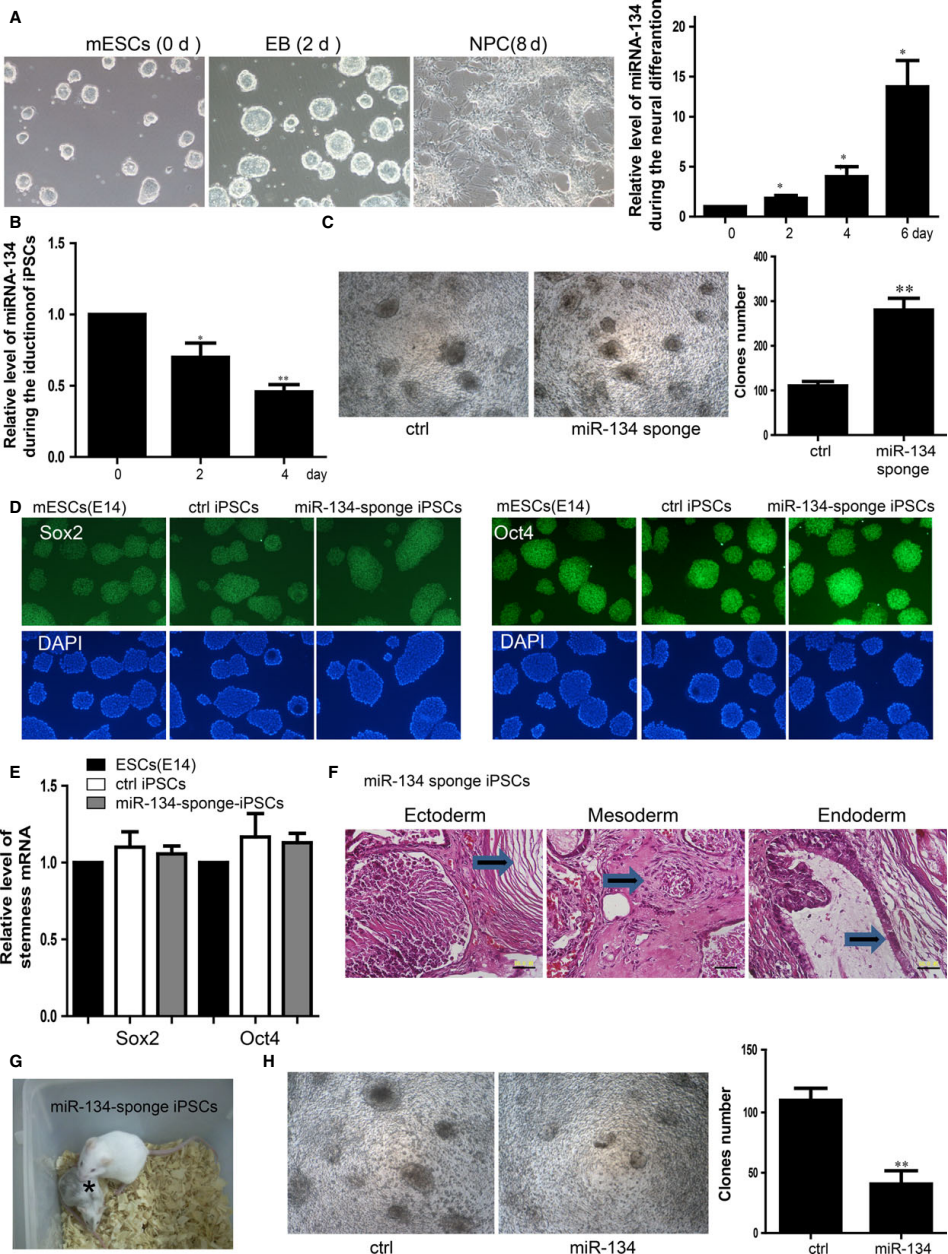
Inhibition of miR‐134 facilitates the initiation of iPSCs generation from Neural Progenitor Cells. (**A**) Induction of the neuralgenesis of NPCs from the mESCs. Right panel showed the expression level of the miR‐134 during the process of induction detected by qRT‐PCR. For all experiments *n* = 3, average ± standard deviation (S.D.), *P < 0.05. (**B**) Expression level of the miR‐134 was down‐regulated during the reprogramming of NPCs. For all experiments *n* = 3, average ± S.D., *P < 0.05, **P < 0.01. (**C**) Inhibition of miR‐134 promoted the reprogramming. The scale bar represents 500 μm. Right panel showed the statistics of clones number. For all experiments *n* = 5, average ± S.D., *P < 0.05, **P < 0.01. (**D**) Immunofluorescent staining showed the expression of Sox2, Oct4 in the mESCs, mature iPSCs and the miR‐134 sponge iPSCs. ctrl iPSCs means the mature iPSCs. The scale bar represents 100 μm. (**E**) qRT‐PCR showed the expression level of Sox2, Oct4 in the mESCs, mature iPSCs and the miR‐134 sponge iPSCs. (**F**) Teratomas derived from miR‐134 sponge‐derived iPSCs. Representative images of haematoxylin and eosin staining for cutin tissue (ectoderm), cartilage or skeletal tissue (mesoderm) and cilium tissue (endoderm) are shown. The scale bar represents 50 μm. (**G**) Two‐week‐old chimeric mice derived from OSKM+miR‐134 sponge‐derived iPSCs (C57BL/6 background) marked by asterisk. (**H**) Clones of the iPSCs overexpressed miR‐134 was less than control group. The scale bar represents 100 μm. Right panel is the statistics of clones. For all experiments *n* = 3, average ± S.D., **P < 0.01. iPSCs, induced pluripotent stem cells.

We performed the induction of neural differentiation from mESCs to NPCs (Fig. 1A) and found that the expression level of miR‐134 was gradually up‐regulated during the 6 days of induction process. We detected miR‐134 expression level during the induction of iPSCs finding that the expression level of miR‐134 was down‐regulated in the reprogramming process of iPSCs derived from NPCs which was taken from the hippocampus of foetal mouse (Fig. 1B). Inhibition of endogenous miR‐134 by miR‐134 sponge which is a complementary strand of miR‐134 promoted the induction efficiency of iPSCs about three times more than control group by detecting the total clones number (Fig. 1C). We further found that the miR‐134 sponge iPSCs showed the similar ability of stemness maintenance as mESCs (E14) as well as the control iPSCs detected expression of stemness genes Sox2 and Oct4 by immunofluorescence staining and qRT‐PCR (Fig. 1D and E). The miR‐134 sponge iPSCs were injected into the dorsal flanks of athymic nude mice (NODSCID) to test their ability to form teratomas. Teratomas were clearly observed at 4 weeks after miR‐134 sponge iPSCs injection, and the subsequent histological analysis showed that the tumours generated by the miR‐134 sponge iPSCs differentiated completely to three layer tissues (Fig. 1F). Furthermore, we performed chimaera generation experiment to test the ability of miR‐134 sponge iPSCs generating chimeric mice. Live chimaeras with black hair contributed by miR‐134 sponge iPSCs were obtained demonstrating that these iPS cells have normal differentiation potential and ability *in vivo* (Fig. 1G). In the contrary, we found that overexpression of miR‐134 repressed the induction of iPSCs (Fig. 1H).

### Inhibition of miR‐134 promotes the maturation of iPSCs

**Figure 2:**
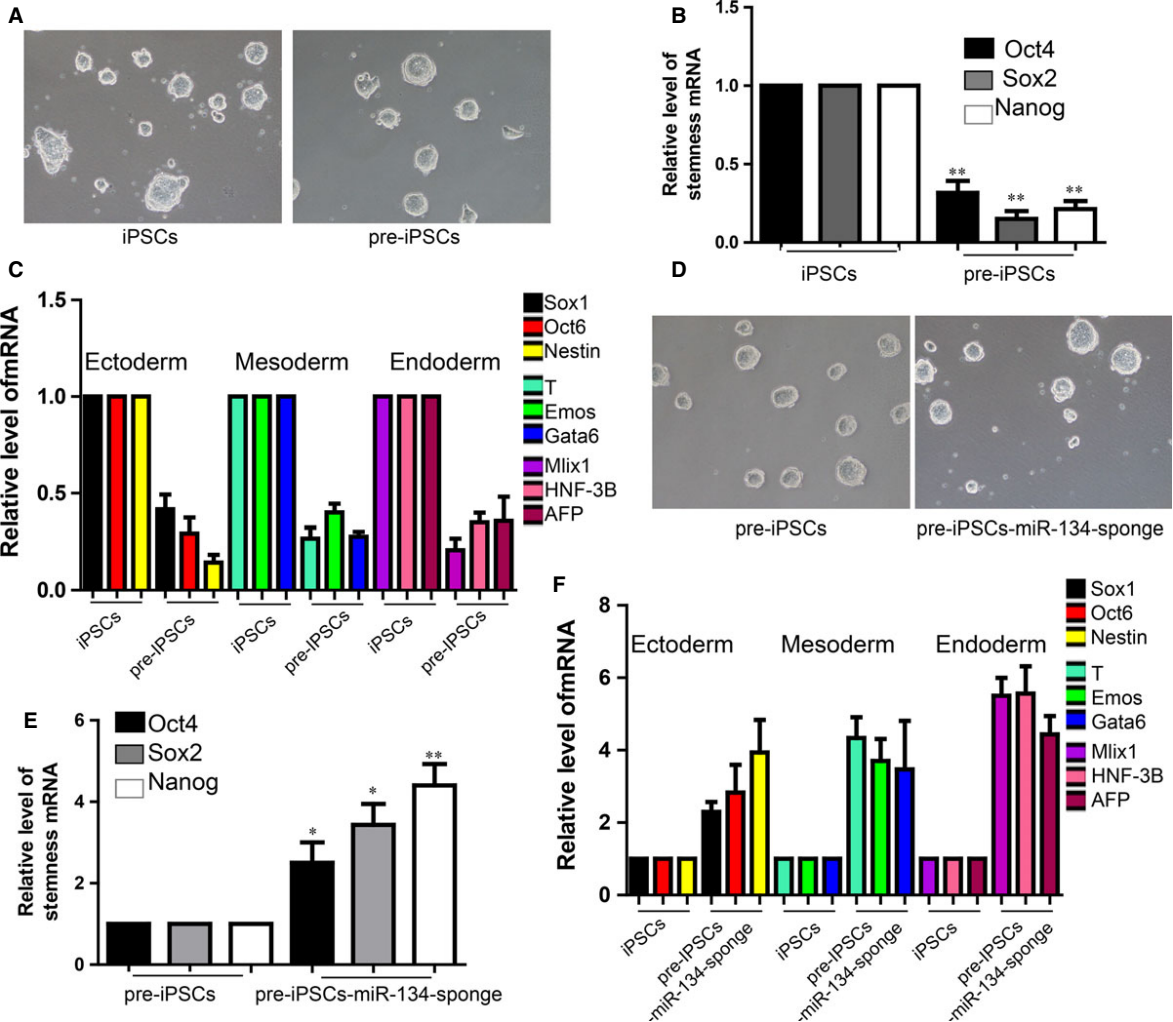
Inhibition of miR‐134 promotes the maturation of iPSCs. (**A**) The morphology of iPSCs and pre‐iSPCs clones. The scale bar represents 100 μm. (**B**) Detection of the expression of stemness markers by qRT‐PCR. For all experiments *n* = 5, average ± standard deviation (S.D.), **P < 0.01. (**C**) Differentiation of the iPSCs and pre‐iSPCs to three germ layer. For all experiments *n* = 5, average ± S.D., **P < 0.01. (**D**) The morphology of pre‐iSPCs overexpressed with miR‐134 sponge. The scale bar represents 100 μm. (**E**) Inhibition of miR‐134 promoted the stemness markers expression in the pre‐iPSCs. For all experiments *n* = 5, average ± S.D., *P < 0.05,**P < 0.01. (**F**) Differentiation of the miR‐134‐sponge‐pre‐iPSCs and pre‐iSPCs to three germ layer. For all experiments *n* = 4, average ± S.D., *P < 0.05, **P < 0.01. iPSCs, induced pluripotent stem cells.

We sorted the pre‐iPSC which is an intermediate and immature state but still have the ability to format clones in the NPCs reprogramming process from somatic cells to iPSCs (Fig. 2A). The expression level of stemness markers were low expressed in pre‐iPSCs than mature iPSCs (Fig. 2B). We also performed the differentiation of pre‐iPSC and iPSCs to three germ layers and found that expression of marker genes were lower in the group of pre‐iPSCs (Fig. 2C). We then overexpressed the miR‐134 sponge into pre‐iPSCs (Fig. 2D) and we found that inhibition of miR‐134 promoted the expression level of stemness markers of Oct4, Sox2, Nanog (Fig. 2E). MiR‐134 sponge overexpressing pre‐iPSCs showed more sufficient ability on differentiation (Fig. 2F). Taken together, our results showed that inhibition of miR‐134 not only enhanced the induction of pre‐ iPSCs but also can push forward the process of maturation of iPSCs.

### MiR‐134 down‐regulated the expression of Mbd3 by directly targeting the 3′ UTR

**Figure 3:**
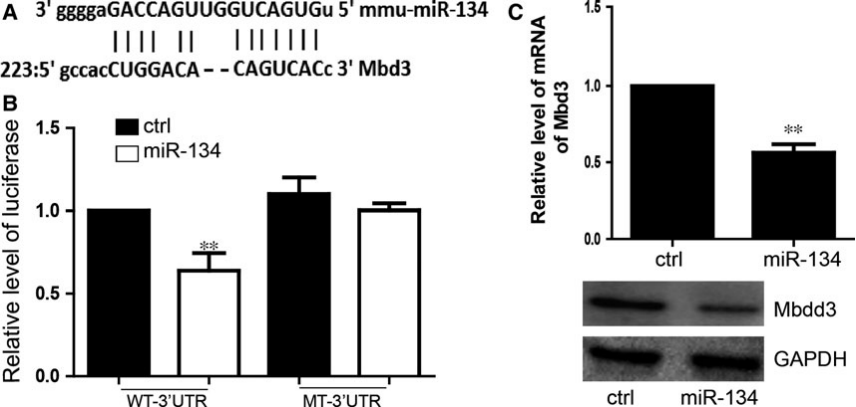
miR‐134 down‐regulated the expression of Mbd3 by directly targeting the 3′ UTR. (**A**) miR‐134 target sites in the 3′UTRs of Mbd3 mRNA are shown aligned with the miR‐134 sequence. (**B**) Luciferase reporter assays were performed. Vectors containing DNA fragments corresponding to the putative wild‐type or mutant target sites for miR‐134 in the 3′UTRs of Mbd3 mRNAs. The luciferase reporter vector was cotransfected with miR‐134 or control miRNA. WT‐3′UTR means the wild‐type 3′UTR. MT‐3′UTR means the mutant 3′UTR which can not bind with miR‐134. For all experiments *n* = 5, average ± standard deviation (S.D.), **P < 0.01. (**C**) miR‐134 repressed the expression of Mbd3 on both mRNA and protein level. For all experiments *n* = 3, average ± S.D., **P < 0.01.

To detect the downstream target of miR‐134, we performed the luciferase reporter assay and found that overexpression of miR‐134 down‐regulated the level of luciferase of wide type 3′UTR of Mbd3. By contrast, there is no significant regulation of miR‐134 on mutant 3′UTR. This study suggested that Mbd3 is a directly target of miR‐134 (Fig. 3A and B). Furthermore, we proved that miR‐134 down‐regulated the expression of Mbd3 on both mRNA and protein level, which indicated that miR‐134, can certainly target to the 3′ UTRs of Mbd3 to repress its expression (Fig. 3C).

### MiR‐134 regulated the iPSCs generation by directly repressing the Mbd3

**Figure 4:**
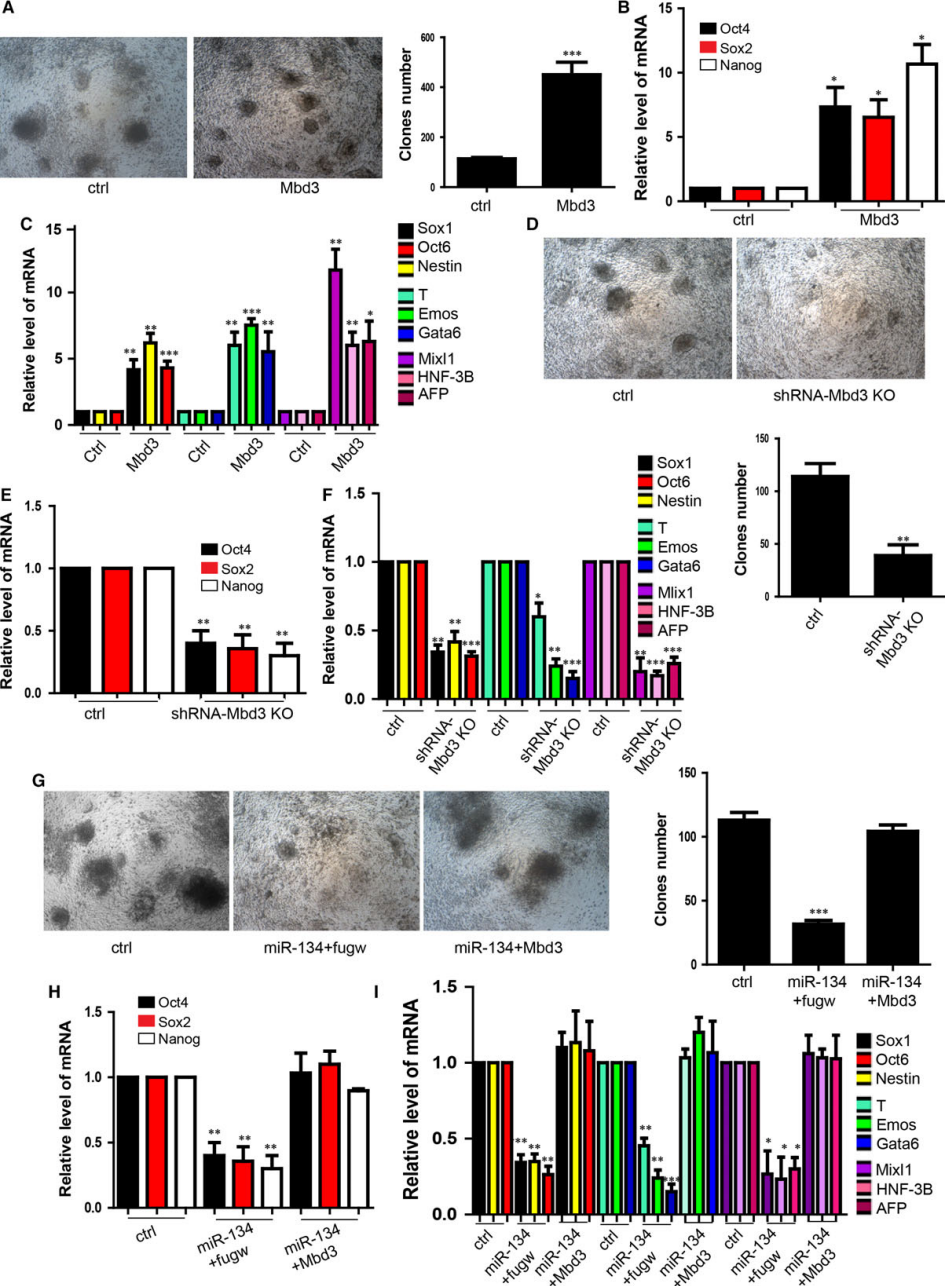
miR‐134 regulated the iPSCs generation by directly repressing the Mbd3. (**A**) The morphology of iPSCs overexpressed with MBD3 and control iPSCs clones. The scale bar represents 500 μm. The bottom panel showed the statistics of clones number. For all experiments *n* = 7, average ± standard deviation (S.D.), ***P < 0.001. (**B**) Detection of the stemness markers Oct4, Sox2, Nanog mRNA level by qRT‐PCR in iPSCs overexpressing Mbd3 or control group. For all experiments *n* = 3, average ± S.D., *P < 0.05, **P < 0.01. (**C**) Detection of the three germ layer markers. For all experiments *n* = 5, average ± S.D., *P < 0.05, **P < 0.01, ***P < 0.001. (**D**) Knockdown of Mbd3 inhibited the reprogramming. The scale bar represents 500 μm. The bottom panel showed the statistics of clone number. For all experiments *n* = 7, average ± S.D., **P < 0.01. (**E**) Stemness markers Oct4, Sox2, Nanog mRNA level in the iPSCs with knockdown of Mbd3 and control group. For all experiments *n* = 4, average ± S.D., **P < 0.01. (**F**) Detection of the three germ layer markers. For all experiments *n* = 3, average ± S.D., *P < 0.05, **P < 0.01, ***P < 0.001. (**G**) Overexpression of Mbd3 rescued the repression of miR‐134 on clones formation. The scale bar represents 500 μm. The right panel showed the statistics of clones number. For all experiments *n* = 7, average ± S.D., ***P < 0.001. (**H**) Overexpression of Mbd3 rescued the repression of miR‐134 on stemness markers mRNA level detected by qRT‐PCR in rescue experiment. For all experiments *n* = 3, average ± S.D., **P < 0.01. (**I**) Overexpression of Mbd3 rescued the repression of miR‐134 on the three germ layer differentiation. For all experiments *n* = 5, average ± S.D., *P < 0.05, **P < 0.01, ***P < 0.001. iPSCs, induced pluripotent stem cells.

To determine whether miR‐134 inhibited reprogramming by targeting Mbd3. We overexpressed Mbd3 in NPCs for further reprogramming and found that there were more clones than control iPSCs (Fig. 4A). The expression level of the stemness markers, Oct4, Sox2, Nanog, were higher in the iPSCs derived from the NPCs which were overexpressed with Mbd3 than control group(Fig. 4B).We also performed the differentiation of the iPSCs and found that iPSCs derived from the NPCs overexpressed with Mbd3 showed higher pluripotency(Fig. 4C). Down‐regulation of Mbd3 by specific small hairpin RNAs (shRNAs) significantly decreased the reprogramming efficiency in comparison with the scrambled control (Fig. 4D). The Mbd3 knockdown iPSCs showed lower capacity of self‐renewal and pluripentcy (Fig. 4E and F).These results also are consistent with the previous study 15. Further we overexpressed Mbd3 and found that Mbd3 blocked the inhibition of miR‐134 on iPSCs induction (Fig. 4G). The function of miR‐134 on inhibiting the capacity of self‐renewal and pluripentcy could be blocked by overexpressing Mbd3 (Fig. 4H and I).

## Discussion

In summary, we uncovered that the miR‐134‐Mbd3 axis can be a regulatory pathway of reprogramming and pluripotency of iPSCs. The functions of miRNAs in iPSCs reprogramming have attracted much attention. miRNAs have been identified and implicated in the regulation of somatic cell‐reprogramming process 18. An increasing number of studies have demonstrated that miRNAs are important in regulating the induction of iPSCs. MiR‐134 was identified to be up‐regulated during RA‐ and N2B27‐induced ectodermal differentiation of mESCs and modulated mouse ES cell differentiation by targeting Nanog and LRH1 and repressing their expression 19. In our study, we found that expression level of miR‐134 is low in self‐renewing mESCs but significantly up‐regulated during neural differentiation and down‐regulated during the induction of iPSCs derived from NPCs. Thus, there might be some unrevealed molecules that strictly control the dynamic expression of miR‐134 during the induction of mature iPSCs. In this study, we used miR‐134 sponge to inhibit the function of the endogenous miR‐134 and found that the iPSCs clone number was increased during this process. Besides, we performed teratoma and chimeric mouse experiments to verify that iPSCs with inhibition of miR‐134 can normally differentiate to the three germ layers and developed to chimeric mouse. In contrast, overexpression of miR‐134 significantly repressed the reprogramming. We further confirmed that miR‐134 can enhance pre‐iPSCs maturation on increasing. We further confirmed that inhibition of miR‐134 increased the expression of stemness related marker genes and promoted the pluripotency of differentiation in pre‐iPSCs. So our data showed that the inhibition of miR‐134 enhanced iPSCs reprogramming meanwhile do not change iPSCs stemness and differentiate capacity. Moreover, pre‐iPSC is an intermediate and immature state with low expression of pluripotency and layer differentiation‐related factors but still have ability to format clones in the process from somatic cells reprogramming to iPSCs. Additionally, inhibition of miR‐134 facilitated the induction of iPSCs generation from NPCs and pushed forward the process of maturation of pre‐iPSCs. These results might provide more insight into the specific roles of miRNAs in reprogramming process to lay the foundation for further clinical application.

In a wide variety of reprogramming processes, miRNAs fine tune or restrict cellular identities by targeting important transcription factors or key pathways to regulate many kinds of biological processes 20, 21. Mbd3 is an essential factor to maintain full mouse ES cell pluripotency by repressing the trophectoderm‐specific differentiation programme 15. Mbd3 function was reported to be dispensable for ES cell growth in culture, but essential for their commitment to a full spectrum of embryonic lineages when aggregated with wild‐type embryos, indicating that pluripotency of these cells is indeed affected 22. Moreover, Mbd3 is critically required for mouse embryonic stem cells both *in vitro* and *in vivo* [22, 23]. Our findings suggested that miR‐134 can bind to the pluripotency‐related factor Mdb3 3′ UTR to repress its expression to play a very significant role in restraining the reprogramming of iPSCs. In our study, we found that miR‐134 directly targeted the Mbd3 3′UTR to down‐regulate its expression. We also found that overexpression of Mbd3 promoted the efficiency of reprogramming. In contrast, down‐regulation of Mbd3 repressed the reprogramming, which is similar with the function of overexpression of miR‐134. To determine whether Mbd3 is the direct downstream mediator of miR‐134 on regulating the reprogramming, we performed the rescue experiments and found that overexpression of Mbd3 blocked the repression of reprogramming caused by miR‐134. These results showed that Mbd3 can be the direct target of miR‐134 and the functional downstream mediator on regulating the reprogramming.

Nanog has an essential role in establishing pluripotent ground state [24], also Nanog can enhance MBD3‐mediated reprogramming of NPCs 15.In our study, we also found that both miR‐134 and MBD3 regulated Nanog expression [19, 25]. Therefore, it was seemed like an alternative explanation that Nanog might be the target of MBD3 and miR‐134 to mediate induced pluripotency. While previous study has also showed that nanog is dispensable for the generation of induced pluripotent stem cells. Nanog–/– mouse embryonic fibroblast (MEFs) or mouse neural progenitor cells (NPCs) can both be reprogrammed to iPSCs. Nanog–/– iPSCs are highly similar to wild‐type pluripotent cells.In the contrary, the Mbd3–/–iPSCs were phenotypically similar to previously reported Mbd3‐null ESCs [26], exhibiting impaired embryoid body (EB) differentiation and slower proliferation 15. Studies also showed that both Mbd3–/– NPCs and Mbd3‐null pre‐iPSCs derived from them exhibited slower proliferation, consistent with previous reports of Mbd3–/– ESCs 15, 22, 23. These studies suggested that nanog could be not the mediator of the function of miR‐134 or Mbd3 during the iPSCs induction.

Our study revealed the miR‐134‐Mbd3 signalling axis on regulating reprogramming efficiency and maturation of iPSCs from NPCs. Investigation of the regulatory mechanism of miR‐134‐Mbd3 signalling axis on induction of pluripotency of iPSCs help elucidate epigenetic mechanisms in iPSCs reprogramming. Our study suggests that we found an important signalling axis which can enhance the reprogramming efficiency and maturity of pluripotency. Finding this miR‐134‐Mbd3 signalling axis helps us for further clinical treatment of iPSCs.

## Conclusion

Inhibition of miR‐134 facilitates the initiation of iPSCs generation and promotes the maturation of iPSCs. And miR‐134 regulated the iPSCs generation by directly repressing the Mbd3. MiR‐134‐Mbd3 axis regulates the induction of pluripotency.

## Author contribution

Conceived and designed the experiments: Lei Zhang, Zhifeng Chen and Hong Jiang. Performed the experiments: Lei Zhang, Ying Zhang and Jia Yan. Analysed the data: Ying Zhang and Yan Jia. Wrote the paper: Lei Zhang and Hong Jiang.

## Conflict of interest

The authors confirm that there are no conflicts of interest.

## Supporting information


**Table S1** qRT‐PCR primer sequence.
