# Supplementary material for: MiR‐134‐Mbd3 axis regulates the induction of pluripotency
Source: J Cell Mol Med. 2016 Feb 29;20(6):1150–8. doi: 10.1111/jcmm.12805 (PMC4882991; doi:10.1111/jcmm.12805)
Supplement: Supplementary file 1 — Table S1 qRT‐PCR primer sequence. [file JCMM-20-1150-s001.docx]

**qRT-PCR primer sequence**

| **Name** | **Sequence** |
| --- | --- |
| Endogenous Oct4 | PF:5’-CCATGCATTCAAACTGAGGCACCA -3’  PR:5’-AGCTATCTACTGTGTGTCCCAGTC -3’ |
| Endogenous Sox2 | PF:5’- GCACGGCCATTAACGGCACAC -3’  PR:5’- CTCCATGCTGTTTCTTGCTGTCCTC -3’ |
| Nanog | PF:5’- AGGACAGGTTTCAGAAGCAGA-3’  PR:5’- CCATTGCTAGTCTTCAACCACTG-3’ |
| Mbd3 | PF:5’-GAAGCTAAGTGGATTGAGTGCC-3’  PR:5’- GACAGCAGCGTCTCATCTGTA-3’ |
| Oct6 | PF:5’-CAGTTCAAGCAACGACGCATC-3’  PR:5’- CGAGAACACGTTACCGTAGAGG-3’ |
| Nestin | PF:5’-CCCCTTGCCTAATACCCTTGA-3’  PR5’-GCCTCAGACATAGGTGGGATG-3’ |
| T(brachyury) | PF:5’-GCTTCAAGGAGCTAACTAACGAG-3’  PR:5’-CCAGCAAGAAAGAGTACATGGC-3’ |
| Eomes | PF:5’- GGCCCCTATGGCTCAAATTCC-3’  PR:5’- GAACCACTTCCACGAAAACATTG-3’ |
| Gata6 | PF:5’-CATCACCATCACCCGACCTAC-3’  PR:5’-GGCCCTGTAAGCTGTGGAG-3’ |
| Mixl1 | PF:5’-ACTGAAGCTAGGTGTTTGAAGC-3’  PR:5’- TCCCAGGAGTCCAACTTTGAG-3’ |
| Hnf-3b | PF:5’-GGAGGCAAGAAGACCGCTC-3’  PR:5’-CCTTTAGCTCGCTTAGGCCAC-3’ |
| AFP | PF:5’-ACCTTCCTGTCTCAGTCATTCT-3’  PR: 5’-CCTGACATCCAGGTAGATTTCCA-3’ |
| GAPDH | PF:5’- TGGCCTTCCGTGTTCCTAC-3’  PR: 5’- GAGTTGCTGTTGAAGTCGCA-3’ |
